# “Luteal Analgesia”: Progesterone Dissociates Pain Intensity and Unpleasantness by Influencing Emotion Regulation Networks

**DOI:** 10.3389/fendo.2018.00413

**Published:** 2018-07-23

**Authors:** Katy Vincent, Charlotte J. Stagg, Catherine E. Warnaby, Jane Moore, Stephen Kennedy, Irene Tracey

**Affiliations:** ^1^Wellcome Centre for Integrative Neuroimaging, FMRIB, Nuffield Department of Clinical Neurosciences, Nuffield Division of Anaesthetics, University of Oxford, Oxford, United Kingdom; ^2^Nuffield Department of Women's and Reproductive Health, University of Oxford, Oxford, United Kingdom; ^3^Oxford Centre for Human Brain Activity, Wellcome Centre for Integrative Neuroimaging, Department of Psychiatry, University of Oxford, Oxford, United Kingdom

**Keywords:** pain, progesterone, analgesia, fMRI, emotion regulation

## Abstract

**Background:** Pregnancy-induced analgesia is known to occur in association with the very high levels of estradiol and progesterone circulating during pregnancy. In women with natural ovulatory menstrual cycles, more modest rises in these hormones occur on a monthly basis. We therefore hypothesized that the high estradiol high progesterone state indicative of ovulation would be associated with a reduction in the pain experience.

**Methods:** We used fMRI and a noxious thermal stimulus to explore the relationship between sex steroid hormones and the pain experience. Specifically, we assessed the relationship with stimulus-related activity in key regions of networks involved in emotion regulation, and functional connectivity between these regions.

**Results:** We demonstrate that physiologically high progesterone levels are associated with a reduction in the affective component of the pain experience and a dissociation between pain intensity and unpleasantness. This dissociation is related to decreased functional connectivity between the inferior frontal gyrus and amygdala. Moreover, we have shown that in the pre-ovulatory state, the traditionally “male” sex hormone, testosterone, is the strongest hormonal regulator of pain-related activity and connectivity within the emotional regulation network. However, following ovulation the traditionally “female” sex hormones, estradiol and progesterone, appear to dominate.

**Conclusions:** We propose that a phenomenon of “luteal analgesia” exists with potential reproductive advantages.

## Introduction

Pregnancy-induced analgesia is well described in animals (1–3) and has also been demonstrated in humans (4, 5). This physiological analgesic state presumably decreases the pain and discomfort that would otherwise be experienced as the gravid uterus distends and exerts pressure on nearby viscera, muscles and nerves. It may also ease the acutely painful experience of labor and delivery. Various mechanisms have been proposed to explain this phenomenon including altered innervation of the cervix and uterus, mechanostimulation of the uterus and cervix, changes in TRPV1 expression and an opioid-mediated pain attenuation (6–10). Induction of pseudopregnancy (producing the hormonal milieu of pregnancy without a developing embryo) in female rats produces opioid-mediated analgesia similar to that seen in pregnancy itself (1) supporting the idea that the analgesia arises, at least in part, secondary to the exponential rise in the sex steroid hormones, estrogen and progesterone, observed in pregnancy.

More modest increases in both these hormones occur on a monthly basis in women with natural ovulatory menstrual cycles. Although estradiol levels rise prior to ovulation, the large increase in progesterone levels is only seen if ovulation has occurred. We were, therefore, interested in whether the high levels of progesterone that occur secondary to ovulation may produce a “luteal analgesia”.

In the context of pregnancy-induced anatomical changes, much of the pain and discomfort is likely to be visceral in origin (relating to the uterus, cervix, bladder, and bowel) and would, therefore, have a greater affective (“unpleasant”) component than pain arising from somatic structures (11, 12). Negative mood induction increases the affective component of the pain experience by disrupting the circuitry involved in emotion regulation (13). Both progesterone and testosterone can influence this circuitry in response to an emotional stimulus, altering both stimulus-related activity (14–16) and functional connectivity between regions of the network (17, 18). To date, no studies have specifically investigated hormonal influences on activity within this emotion regulation network in response to experimental pain although the reactivity of the amygdala in response to a noxious stimulus does vary in association with both estradiol (19) and testosterone (20).

Here, we investigated the influences of endogenous levels of sex steroid hormones on pain-related activity and functional connectivity within a network involved in emotion regulation in healthy women. We restricted our investigations to women in an endogenous high estradiol state, and compared those with concomitant low and high levels of progesterone. We hypothesized that a high progesterone level would be associated with a reduction in behavioral measures of pain, particularly the pain unpleasantness. We expected that stimulus-related activity within the emotional regulation network and functional connectivity between regions of this network would be related to serum hormone levels. Moreover, we expected that alterations in activity and functional connectivity within this network would relate to changes in subjective reports of pain unpleasantness.

## Materials and methods

### Subject recruitment and selection

The Central Oxfordshire Research Ethics Committee provided ethical approval for this study and written informed consent was obtained from all subjects in accordance with the Declaration of Helsinki. Our aim was for 12 women to complete the study. Subjects were recruited by advertisement and word of mouth. They were required to have regular menstrual cycles, not to have used centrally acting medication or exogenous hormones for the preceding 6 months and not to have dysmenorrhea (defined as pain with menstruation ≥3/10) or any other chronic pain condition. Subjects completed a detailed medical questionnaire to ensure they were eligible to participate in the study. This included numerical rating scales for pain with menstruation and at other times throughout the cycle; the Rome III criteria for Irritable Bowel Syndrome (IBS) (21), and detailed questions relating to bladder function, including symptoms associated with bladder filling and emptying, to allow identification of possible interstitial cystitis/painful bladder syndrome. All subjects underwent screening to ensure they did not meet any of the exclusion criteria for MR experimentation such as the presence of implanted devices (including pacemakers, aneurysm clips etc.), recent surgery, previous injury involving metal, non-removable body piercings and pregnancy.

Thirteen women were recruited and 12 completed all parts of the study. The subject who withdrew commenced hormonal contraception before her second scan could be scheduled.

### Noxious stimulation

A thermal resistor (developed in-house) was used to deliver noxious thermal stimuli as previously described (20, 22–25). Stimuli were delivered to the left inner forearm and the midline lower abdomen (T10-12). Only data from the arm stimulation were investigated here.

### Experimental design

We aimed to optimize endogenous hormonal variation and therefore the timing of experimental sessions was anchored around the menstrual cycle. A high estradiol, low progesterone state is expected immediately prior to ovulation and thus one session was scheduled for day 10–12 of the cycle (where day 1 is the first day of menstruation). The highest levels of progesterone are seen in the mid-luteal phase and thus a second session was scheduled for day 20–22 of the cycle, and women used ovulation kits (InstAlert, Acon Laboratories, San Diego, USA) in this cycle to confirm occurrence of ovulation. A further session was performed on day 1–2 when both estradiol and progesterone should be low. The order of these sessions was randomized across the group.

Immediately before each scan, subjects completed a state anxiety (26) and current pain questionnaire. Thermodes were placed as described above, and the subject positioned in the scanner. A modified random staircase method was used to identify the temperature at each site that corresponded to a pain intensity rating of 5/10 (0 = no pain, 1 = just painful, 10 = extremely painful). During the functional scans, 10 stimuli at this temperature, each lasting 3 s, were delivered with an inter-stimulus interval of 55–65 s. Twelve seconds after the termination of each heat stimulus a visual analog scale (VAS) was displayed on the screen for 9 s, with anchors “no pain” and “extremely painful”. After 1 s, a further VAS with anchors “not unpleasant” and “extremely unpleasant” was displayed for 9 s. Subjects rated the pain intensity and unpleasantness of each stimulus with a slider using their right hand. The order in which the two sites were stimulated was randomized across the group.

Blood for a hormone profile was drawn from an antecubital vein immediately after completion of each scanning session.

In their own time, subjects also completed a detailed medical, gynecological and obstetric questionnaire. This included a set of validated psychological tools: trait anxiety (26), Beck Depression Inventory (BDI) (27), Pain Catastrophising Scale (PCS) (28) and Pain Vigilance and Awareness Questionnaire (PVAQ) (29), and a quality of life measure, the SF-36 (30).

### fMRI data acquisition

Subjects were scanned in a 3 Tesla Siemens/Varian MRI system with a bird-cage radio frequency coil and a four channel phased-array receiver coil. A standard whole-brain gradient echo-planar imaging (EPI) sequence was used for the 3 functional scans (repetition time (TR) = 3 s; echo time (TE) = 30 ms; 3.5 mm thick axial slices; 200 volumes (the first four are “dummy” scans), field of view (FOV) = 224 × 224 mm, matrix = 64 × 64 × 41, voxel size = 3.5 × 3.5 × 3.5 mm). A field-map was also acquired with the same parameters to aid accurate registration. In addition, at one visit a T1-weighted structural scan (64 slices × 3 mm) was taken for anatomical overlay of activation.

### Analysis of serum hormones

As previously described (20, 25), blood samples were centrifuged for 10 min at 1,300 rpm, and serum was extracted and stored at −80°C for batch analysis of samples by Pfizer Laboratory, New Haven, USA, with commercially available assays (Axsym, Abbott Laboratories, Illinois, USA). Total (i.e., free and protein-bound) serum concentrations were assayed using a microparticle enzyme immunoassay (MEIA) technology for the sex steroid hormones estradiol, progesterone and testosterone, and a fluorescence polarization immunoassay (FPIA) for cortisol.

### Statistical analysis

#### Choice of data sets for analysis

As described previously (20), despite careful scheduling of scans with respect to the first day of menstruation and the use of ovulation kits, hormone profiles were not all as expected for the appropriate menstrual cycle phase. This is consistent with literature describing marked inter- and intra-individual variation in endogenous hormone levels even in healthy parous women (31). In order to address our hypothesis, data were categorized for further analysis on the basis of hormone levels. Two groups were therefore selected from the 36 data sets obtained with hormonal profiles representative of the two distinct biological states: pre- and post-ovulation. High estradiol was defined as described previously as >51 pg/mL (20), whilst the level of progesterone considered representative of ovulation [>1.0 ng/mL (32)] was chosen to define high progesterone. The two groups selected for further analysis were: those with a high estradiol and low progesterone (“LowP”: E2 > 51 pg/ml; *P* ≤ 1.0 ng/mL; 11 data sets) and those with high levels of both estradiol and progesterone (“HighP”: E2 > 51 pg/ml; *P* > 1.0 ng/mL; 10 data sets). The LowP group comprised data from nine individuals (two sampled at two time points), whilst the HighP group included data from eight individuals (two sampled at two time points). All subsequent statistical analyses accounted for the fact that each group contained repeated measures data for two individuals. Moreover, the two groups were not considered independent due to the fact that most subjects were represented in both the LowP and HighP groups and repeated measures were appropriately modeled.

#### Psychophysical data

Psychophysical data were analyzed with SPSS version 22 (SPSS Inc., Chicago). Serum estradiol, progesterone and testosterone levels were not normally distributed and were therefore log-transformed prior to analysis. An additional variable was created for the dissociation between pain intensity and unpleasantness by subtracting unpleasantness ratings from intensity ratings. Linear mixed models were used to investigate the effect of group on the data, taking into account repeated measures and the unbalanced size of the groups. A *p* < 0.05 was considered significant. Pearson's partial correlations were used to investigate relationships between behavioral data and hormone levels, controlling for repeated measures, after confirming that data were normally distributed.

#### Imaging data

All analyses were performed using FEAT (FMRIB Expert Analysis Tool) Version 5.98, part of FSL (FMRIB's Software Library; www.fmrib.ox.ac.uk/fsl) (33).

#### Analysis of stimulus evoked responses

The following pre-processing steps were applied to each set of FMRI data: removal of the first four dummy volumes, removal of non-brain signal using a Brain Extraction Tool (BET) (34), motion correction (35), B0 field unwarping (36), spatial smoothing using a Gaussian kernel of full-width-half-maximum of 5 mm, demeaning of each voxel time course, and nonlinear high-pass temporal filtering (cutoff: 90 s). A general linear modeling (GLM) approach was used to model the response to thermal stimuli. The stimulus input function was convolved with a gamma hemodynamic response function (standard deviation: 3 s, mean lag: 6 s) to generate the regressor of interest. The estimated motion parameters for each subject were included as covariates of no interest to reduce spurious activations due to head motion. Registration was performed to the subject's T1 high-resolution structural image and then to standard space (Montreal Neurological Institute (MNI) 152 brain) using FLIRT (FMRIB's Linear Image Registration Tool) (37).

All higher-level analysis was carried out using FLAME (FMRIB's Local Analysis of Mixed Effects) stage 2 with automatic outlier detection (38, 39). Results were considered significant if *Z* > 2.3, with a cluster threshold of *P* < 0.05 corrected for multiple comparisons. Firstly the main effect of noxious stimulation was assessed for the two groups separately by obtaining average group responses to noxious thermal stimulation (activations and deactivations). The low and high progesterone groups were then formally compared with a *t*-test, including additional explanatory variables of no interest to account for repeated measures.

#### Analysis of hormonal influences on the emotion regulation network

To specifically investigate the relationship between sex steroid hormones and the emotion regulation network in the context of painful stimuli, we investigated both activity within relevant regions of interest (ROI) and functional connectivity between these ROIs (40). The four ROIs chosen for these analyses were (1) the amygdala; (2) the orbital frontal cortex (OFC); (3) the inferior frontal gyrus (IFG); and (4) the nucleus accumbens (NAc). The amygdala was chosen due to its central role in emotion and pain processing. The OFC was chosen because of its key role in emotion regulation and the known hormonal influences on OFC-amygdala connectivity in response to other emotional stimuli (18). The IFG, a sub-region of the ventrolateral PFC (vlPFC), was chosen due to its relationship with altered unpleasantness after depressed mood induction (13) and its proposed role in cognitive reappraisal of negative emotions (41). Finally, the NAc was chosen as it has been suggested that negative reappraisal is mediated through a pathway from the PFC via the amygdala, whilst positive reappraisal is from the PFC via the NAc (42). The Harvard-Oxford Cortical and Subcortical Structural Atlases were used to define these ROIs with a threshold of 50%. Activity was obtained by extracting the mean percentage BOLD signal change in each ROI in response to noxious stimulation.

We specifically explored connectivity between the OFC and the amygdala; the OFC and the NAc; the IFG and the amygdala; and the IFG and the NAc. To reduce the number of comparisons performed, analyses were limited to the left hemisphere (ipsilateral to the noxious stimulus) because (i) in the whole brain analyses the greatest differences between the hormonal states was seen on the ipsilateral side; and (ii) emotion regulation circuitry activated by left sided noxious stimuli in a depressed mood condition has previously been shown to be predominantly ipsilateral (13). However, we acknowledge that issues surrounding lateralization in emotion processing are complex (43). Functional connectivity was calculated by first extracting the mean timecourse of activation in each region across the whole paradigm, and then correlating the two timecourses using Pearson's correlation. As Pearson's correlation coefficients are not usually normally distributed, the resulting correlation coefficients were Fisher transformed prior to further analysis. The relationship between the three steroid hormones and these measures of activity and connectivity were assessed in both the low and high progesterone state, using Pearson's partial correlation to control for repeated measures.

## Results

There was no significant difference in plasma levels of any of the hormones other than progesterone between the groups (Table 1). Across all 21 high estradiol data-sets, there were no correlations between each of the four hormone levels. When the HighP group was considered separately, estradiol and progesterone levels were correlated (Pearson's partial correlation, controlling for repeated measures: *r* = 0.801, *p* = 0.01), but no other correlations were seen in either group.

**Table 1:** Serum hormone profiles.

**Hormone**	**LowP (11 data sets)**	**HighP (10 data sets)**	**Significance**
Estradiol (pg/mL)	134 ± 22	103 ± 12	NS
Progesterone (ng/mL)	0.33 ± 0.09	8.74 ± 2.20	*p* = 0.001
Testosterone (pg/mL)	280 ± 27	230 ± 26	NS
Cortisol (mcg/dL)	9.2 ± 1.6	8.2 ± 1.1	NS

*Only data-sets where estradiol levels were high (>51 pg/mL) were included, these were subdivided on the basis of progesterone levels into low (LowP; progesterone <1.0 ng/mL) and high (HighP; progesterone >1.0 ng/mL) groups. Data are presented as means ± SEM. Two-tailed linear mixed models were used to assess for an effect of group, taking into account repeated measures and the unbalanced size of the groups*.

### High progesterone levels are associated with lower pain unpleasantness ratings

**Figure 1:**
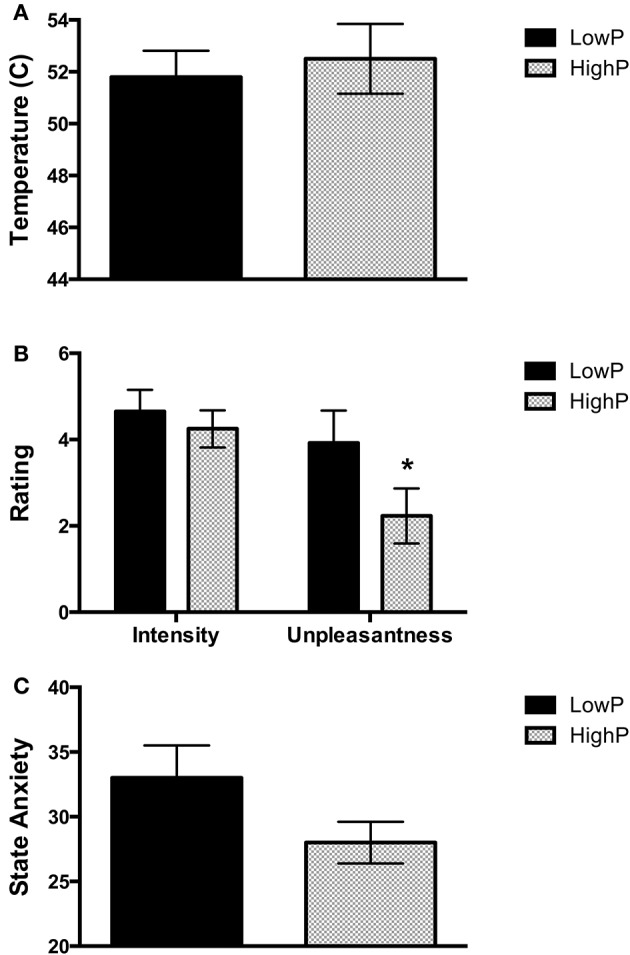
Behavioral measures obtained in low and high progesterone states. **(A)** Temperature required on the left inner arm to obtain a pain intensity rating of 5 out of 10. There was no significant difference in the temperature required between the groups. **(B)** Pain intensity and unpleasantness ratings in response to repeated stimuli at this temperature. Women in a high progesterone state rated the stimuli as significantly less unpleasant than those in a low progesterone state (*p* = 0.032), but there was no significant difference in pain intensity ratings. **(C)** State Anxiety levels. There was a trend toward a difference in state anxiety levels between the two groups (*p* = 0.053). Data are expressed as mean ± SEM. One-tailed linear mixed models were used to assess for an effect of group (^*^p < 0.05). P, progesterone.

Supporting our hypothesis, pain unpleasantness ratings in response to the noxious stimuli (which reflect the affective component of pain) were significantly lower in the HighP compared to the LowP group (2.23 ± 1.80 [mean ± S.D] vs. 4.25 ± 2.26, F_(1, 19)_ = 3.87, *p* = 0.032). There were no significant differences between the groups in the other pain measures [i.e., the temperature required to elicit a pain intensity rating of 5/10 (52.5 ± 3.81 vs. 51.8 ± 3.04°C) and pain intensity ratings (3.92 ± 1.23 vs. 4.65 ± 1.52)]. State anxiety scores showed a trend toward lower values in the HighP group, reflecting lower anxiety at that scan point (27.9 ± 4.5 vs. 33.4 ± 7.6), but this did not reach statistical significance [*F*_(1, 19)_ = 2.90, *p* = 0.053; Figure 1].

We correlated also the hormone levels and behavioral measures across all the high estradiol datasets (*n* = 21). As expected, progesterone levels were significantly related to pain unpleasantness ratings [*r*_(18)_ = −0.494, *p* = 0.027], such that higher progesterone was associated with a lower affective pain component. Testosterone levels were significantly related to the temperature required to produce a fixed pain intensity [*r*_(18)_ = −0.449, *p* = 0.047], such that lower temperatures were required with higher testosterone levels. There was no significant relationship between estradiol level and any of the measures obtained (i.e., temperature, pain intensity, pain unpleasantness and anxiety).

**Figure 2:**
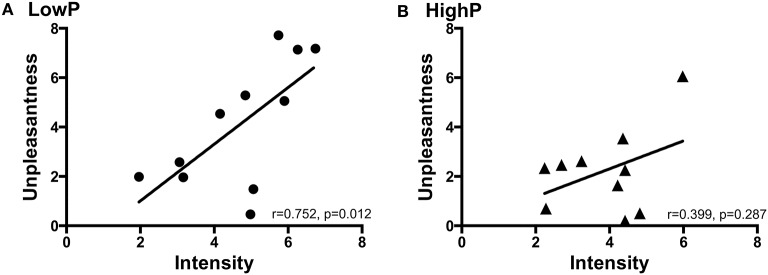
The relationship between pain intensity and unpleasantness ratings in **(A)** LowP state and **(B)** HighP state. Pearson's partial correlation was used to investigate the relationship controlling for repeated measures. Pain intensity and unpleasantness ratings were significantly positively correlated in the low P state [*r*_(8)_ = 0.752, *p* = 0.012]. There was no significant correlation between pain intensity and unpleasantness ratings in the HighP state. P, progesterone.

In line with the hypothesis that higher progesterone levels may modulate the affective component of pain perception without modifying the intensity of that pain, there was a strong correlation between pain intensity and unpleasantness in the LowP group [*r*_(8)_ = 0.752, *p* = 0.012] but no significant relationship between the two ratings in the HighP group [*r*_(7)_ = 0.399, *p* = 0.287; Figure 2]. However, there was not a statistically significant difference between these relationships.

### High progesterone is associated with reduced activation within the emotion processing network in response to painful stimuli

**Figure 3:**
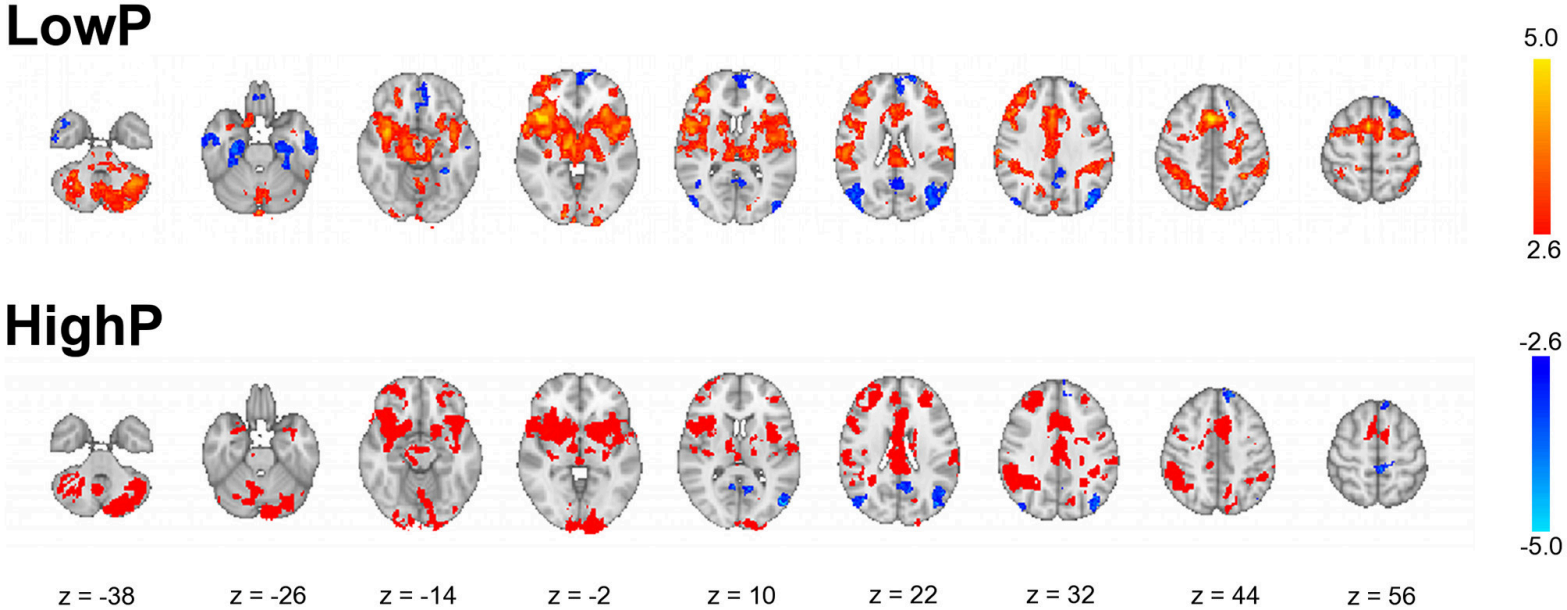
Results of whole brain mixed-effects analyses of the average group response to noxious thermal stimulation of the left inner arm. The data shown are the results of mixed-effects analyses with outlier de-weighting and are corrected for multiple comparisons, *Z* > 2.3, *p* < 0.05. Images shown are thresholded at *Z* > 2.6 for illustration and are representative. Images are presented radiologically, with red-yellow representing activation and blue deactivation.

**Figure 4:**
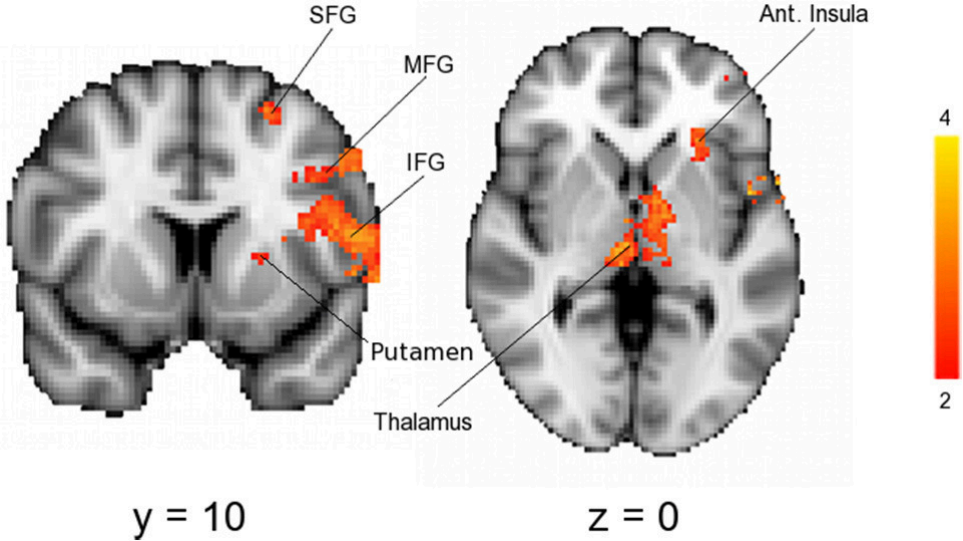
Results of a mixed-effects analysis comparing brain activation in response to noxious stimulation of the left arm in a low compared to a high progesterone state (i.e., lowP > highP). Data shown are the results of mixed-effects analyses with outlier de-weighting and are corrected for multiple comparisons, *Z* > 2.3, *p* < 0.05. No brain areas were more active in the opposite contrast, HighP—LowP. Areas of activation are shown in Table 2. Images shown are *y* = 68 and *z* = 36 and are representative. Images are presented radiologically. SFG, superior frontal gyrus; MFG, middle frontal gyrus; IFG, inferior frontal gyrus.

As expected, across the whole group noxious stimuli led to activation in regions known to be involved in the central processing of pain (44) (Figure 3). In line with the hypothesis that high progesterone levels lead to analgesia, when the response to noxious stimuli was compared between the groups, greater activation in the LowP group than the HighP group was seen in the contralateral primary somatosensory cortex (S1), bilateral thalamus and ipsilateral frontal gyri, insula, putamen and amygdala (Figure 4; Table 2). No brain regions were more active in the reverse contrast (i.e., highP > lowP).

**Table 2:** Brain regions where significantly greater pain-related activation was seen in LowP compared to HighP groups.

**Region**	**Max Z Statistic**	**Side**	**MNI Co-ordinates**		
			***x***	***y***	***z***
M1^*^	3.34	Bilateral	2	−44	58
S1	3.49	R	34	−30	54
Superior frontal gyrus	3.28	L	−24	2	52
Middle frontal gyrus	3.21	L	−54	8	38
Inferior frontal gyrus	3.56	L	−54	14	18
Anterior Insula	3.27	L	−30	20	8
Putamen	2.93	L	−26	14	6
Thalamus	3.20	L	−14	−24	10
Thalamus	3.39	R	8	−20	0
Amygdala	2.45	L	−16	−10	−10

*Results are expressed as peak z scores derived from a mixed-effects analysis with outlier de-weighting and a cluster threshold at Z > 2.3, p < 0.05*.

*M1, primary motor cortex; S1, primary somatosensory cortex*.

* *Represents regions where the difference was driven by greater deactivation in HighP state*.

### High progesterone is associated with decreased connectivity within the emotion regulation network

The relationship between hormone levels and both activity and functional connectivity within the network were different between the low and high progesterone states (Table 3). In line with the hypothesis that higher levels of progesterone modulates the brain's affective response to pain, in the highP group, the traditionally “female” hormones, estradiol and progesterone were related to both activity within the selected ROIs and functional connectivity between them. Progesterone levels were negatively related to activity in the amygdala and OFC, and to functional connectivity between the IFG and amygdala. Estradiol levels were negatively related to amygdala activity and to connectivity between the OFC and amygdala but positively related to connectivity between the OFC and NAc.

**Table 3:** Hormonal influences on the emotion regulation network.

	**Low P**			**High P**		
		***r***	***p***		***r***	***p***
**ESTRADIOL**						
Activity				Amygdala	−0.670	0.048
Connectivity				OFC-Amygdala	−0.799	0.010^*^
				OFC-NAc	0.801	0.009^*^
**PROGESTERONE**						
Activity				Amygdala	−0.687	0.041
				OFC	−0.674	0.046
Connectivity				IFG-Amygdala	−0.842	0.004^*^
**TESTOSTERONE**						
Activity	Amygdala	−0.641	0.046			
	OFC	−0.689	0.027			
Connectivity	OFC-NAc	−0.697	0.025			

ROI analyses were performed to establish whether serum hormone levels related to activity within and connectivity between four key regions of the emotion regulation network. Pearson's partial correlations were performed to investigate the relationships controlling for repeated measures. All relationships significant at p < 0.05 are shown; those that survive correction for multiple comparisons (four ROIs: p < 0.05/4 = 0.0125; four connectivity relationships: p < 0.05/4 = 0.0125) are highlighted with an asterisk.

*P, progesterone; OFC, orbitofrontal cortex; IFG, inferior frontal gyrus; NAc, nucleus accumbens*.

However, for subjects in a low progesterone state only testosterone was found to have a significant relationship with either activity or connectivity of the regions investigated. Specifically, serum testosterone levels were negatively correlated with activity in the amygdala and the OFC, and to connectivity between the OFC and NAc. The relationships between the hormones and functional connectivity in the HighP state survived correction for multiple comparisons, however, this was not the case for the relationships with activity, nor for any of the relationships in the lowP state.

### Dissociation between pain intensity and unpleasantness is related to decreased functional connectivity between the IFG and amygdala

**Figure 5:**
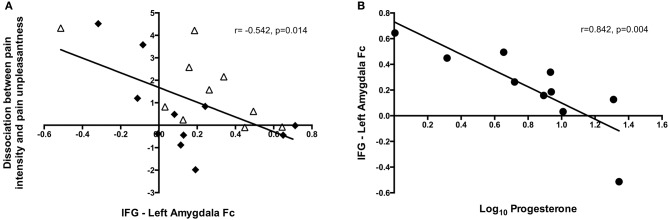
Insights into the neural mechanisms underlying the dissociation between pain intensity and unpleasantness. **(A)** The amount of dissociation between pain intensity and unpleasantness in a high estradiol state was negatively correlated with the strength of functional connectivity between the IFG and left amygdala [*r*_(18)_ = −0.542, *p* = 0.014]. Black diamonds represent measures obtained in the LowP state and open triangles those obtained in a HighP state. **(B)** In a high progesterone state, functional connectivity between the IFG and left amygdala was correlated with serum progesterone levels [*r*_(7)_ = −0.842, *p* = 0.004]. Pearson's partial correlation was used to investigate the relationship controlling for repeated measures.

To investigate the neural mechanisms underlying the observed dissociation between pain intensity and unpleasantness in response to higher progesterone levels, we subtracted pain unpleasantness ratings from pain intensity ratings. Factors relating to this dissociation were then explored. Across all the high estradiol datasets there was a trend toward a relationship between progesterone levels and the degree of dissociation [*r*_(18)_ = 0.420, *p* = 0.065], such that higher progesterone levels were associated with greater dissociation. This dissociation was not significantly related to activity in any brain regions investigated, but was negatively correlated with functional connectivity between the IFG and amygdala [*r*_(18)_ = −0.542, *p* = 0.014; Figure 5A].

In the HighP group there was a strong relationship between serum progesterone levels and IFG-Amygdala connectivity [*r*_(7)_ = −0.842, *p* = 0.004; Figure 5B], such that higher progesterone was associated with decreased functional connectivity. This was significantly different (*Z* = −3.19, *p* = 0.0014) from the situation in the LowP group where there was no relationship between progesterone levels and connectivity.

## Discussion

In this study we used an experimental paradigm combining fMRI with a noxious thermal stimulus, behavioral measures and serum hormone profiles to investigate the influence of sex steroid hormones on pain-related activity and functional connectivity within circuitry involved with emotion regulation. We have demonstrated the existence of a luteal analgesia, which is in line with the other hormonally-driven analgesic states of stress-induced analgesia (45) and pregnancy-induced analgesia (5). This state of reduced pain unpleasantness occurs in association with the high levels of progesterone produced by the corpus luteum after spontaneous ovulation in healthy women. We have shown that the dissociation between pain intensity and unpleasantness that occurs with physiologically high levels of progesterone is related to decreased functional connectivity between the IFG and amygdala. Moreover, we have demonstrated that in the pre-ovulatory state, the traditionally “male” sex hormone, testosterone, is the strongest hormonal regulator of pain-related activity and connectivity within the emotional regulation network. However, following ovulation the traditionally “female” sex hormones, estradiol and progesterone, appear to dominate.

### Pain as an emotional stimulus

It is well accepted that pain is an emotional as well as a sensory experience (46). In agreement with previous studies (44, 47), we have shown that noxious thermal stimulation in both the LowP and HighP states alters brain activity in regions associated with the processing of emotions and emotion regulation (Figure 3). A number of previous studies have shown that modulating the emotional state alters the subjective experience of pain and patterns of pain-related brain activity (13, 23, 48–51). Here, we demonstrate that in healthy women the affective component of pain (“pain unpleasantness”) can be reduced in association with altered activity and functional connectivity within the emotion regulation network without intentionally manipulating mood. It is interesting to note that the hormonal influences we demonstrate on activity and functional connectivity within the emotion regulation network in response to noxious stimuli (traditionally considered a sensory stimulus) are similar to those observed in response to emotional stimuli without a sensory component (52, 53). Moreover, these findings are similar to those found in a study of hormonal influences on the reward system (54), suggesting that these relationships may impact on a wide range of behaviors.

Considering pain as an emotional stimulus, the relationship between estradiol and the functional connectivity of the OFC is particularly notable. Here, we have shown that when both estradiol and progesterone are high, higher estradiol is associated with greater functional connectivity between the OFC and the NAc, and decreased functional connectivity between the OFC and the amygdala (Table 3). In the context of previous work (42), this finding would suggest that the high estradiol levels that persist after ovulation favor positive reappraisal of an emotional stimulus.

It is relatively unusual to see a dissociation of pain intensity and unpleasantness ratings in response to a noxious somatic stimulus, without employing a specific psychological (13, 50, 55) or pharmacological (56) intervention. Whilst our sample size was not large enough to demonstrate a significant difference between these relationships in a high and low progesterone state, the potential therapeutic implications of such a dissociation suggest that future studies should both aim to replicate this finding and explore whether providing exogenous progesterone has similar effects to endogenous fluctuations. If so, there may be potential to consider the use of progesterone as a therapeutic option to reduce the unpleasantness of either acute or potentially chronic pain. This may be of particular value in visceral procedures (e.g., colonoscopy, hysteroscopy) and pain conditions (e.g., Irritable Bowel Syndrome, IBS, Painful Bladder Syndrome, PBS).

### Influence of the hormonal milieu on pain modulatory factors

A particularly notable finding from this study is the influence that the hormonal milieu has on the factors influencing the pain experience. Thus, within the regions investigated, testosterone appears only to influence pain-related activity at times of low progesterone; whilst for estradiol (given the constraint that the individual is in a high estradiol state already) such a relationship is only seen when progesterone levels are high. There is increasing evidence that the influence of one hormone on brain function depends on the level of the other(s) (20, 54, 57, 58) and this observation may contribute to the contradictory literature surrounding the relationship between hormones and pain (59). Even studies investigating hormonal influences on emotional stimuli, where the relationships have been far better characterized than they have in the context of pain (52), have focused on women in a low estrogen state. Therefore, little is known about the relationship between hormones and the response to any emotional stimuli in a high estrogen state, despite the fact that endogenous estrogen levels are high for approximately two-thirds of the natural menstrual cycle.

Whilst adding to the literature by detailing the influences of these hormones on the emotion regulation network in the two physiological hormonal states that occur at times of high estradiol, the findings from the study presented here do not give insights into the mechanisms by which these may be generated. There are two plausible explanations, which are not mutually exclusive. Firstly, one hormone may be required to prime the neuron before a second can exert its effect. This could potentially occur by either a traditional genomic mechanism or be a non-genomic rapid effect (60). Alternatively, alterations in resting state activity (61, 62) or neurotransmitter tone secondary to the hormonal environment could influence the amount of further activation or deactivation possible in response to an experimental stimulus. The low endogenous opioid tone previously demonstrated in a low estrogen and low progesterone state (19) may contribute to revealing influences of other factors on the pain experience that are not able to exert an effect when endogenous opioid tone is high. Combined fMRI/PET and spectroscopy studies would allow an investigation of the relative extent to which modulation of GABA [influenced by both estradiol and progesterone 63) and endogenous opiate activity underlie the hormonal influences on pain.

It is important to remember that this study investigated women who were both physically and psychologically healthy and that all volunteers were carefully screened to ensure that they had no chronic pain, including dysmenorrhea. It has previously been shown that the interaction between occipital cortex GABA levels and both estradiol and progesterone (and its metabolites) is altered by the presence of psychological morbidity (premenstrual dysphoric disorder) (63). Moreover, the presence of chronic pain has been shown to alter pain-related activation of the PFC (64–66), NAc (67) and amygdala (68, 69), and studies investigating hormonal influences on pain appear to produce more consistent results in women with chronic pain conditions (59). Therefore, the presence of either chronic pain itself or psychological morbidity associated with chronic pain may alter the influences of sex steroids on the emotion regulation network. It is important that these findings are replicated in women with chronic pain before starting to consider whether there may be any therapeutic benefit of inducing a high estradiol/high progesterone state. However, a recent study demonstrating a negative relationship between daily progesterone levels and pain ratings in women with fibromyalgia is in line with our findings (70).

Although not significant, we did identify a trend toward lower anxiety levels in the high progesterone state. This is consistent with animal and human studies suggesting that both progesterone and its metabolites have anxiolytic activity (71–74) and some data supporting an associated reduction in pain (71). Given that increased anxiety is known to amplify pain in both healthy subjects (23, 75) and chronic pain patients (76–78), such an association is not surprising. However, as hippocampal regions are known to be involved in pain amplification associated with anxiety (23) and have also been shown to be modulated by progesterone and its metabolites (15, 62, 79), future studies in this area should include an exploration of these brain regions too.

Since the 1960s there has been increasingly widespread use of exogenous hormones as both contraceptives and treatments for gynecological and dermatological conditions. Many of these preparations aim to induce an anovulatory state and thus maintain circulating estradiol and/or progesterone at relatively low levels. In the context of our findings, it is interesting to speculate on the effect this may have on both pain perception and emotional responses more generally. In particular, maintaining a low estradiol state as is seen in both combined oral contraceptive users and those using high dose progesterone preparations may reduce the opportunity for positive reappraisal of stimuli. Given how commonly used these preparations are, it would be worthwhile future studies exploring these issues and the longer-term implications in more detail.

## Limitations of the study

The major limitation of this study is the small sample size of the subgroups (11 and 10 data sets) and that unfortunately not every subject had a high estradiol/low progesterone session and a high estradiol/high progesterone session to allow a direct comparison. The experiments were carefully timed around the menstrual cycle, using ovulation kits as recommended (80), with the expectation that each subject would have a session in both these hormonal states. All the serum samples were frozen and then batch analyzed once the study was complete to avoid introducing error and therefore it was not possible to add additional subjects after the analysis. Our statistical analyses were carefully designed to model the repeated measures and unbalanced group sizes that this situation produced. Although it would have increased our sample size, we did not compare the women in the two phases of their cycle as the specific aim of this study was to assess hormonal influences not cycle phase effects. Previously, carefully controlled cycle phase has been shown to influence the brain response to noxious stimulation, not all of which could be explained by hormone levels (81). However, this study related brain activation to changes in hormone levels between the phases not to the absolute levels of the hormones. As discussed above, we consider it important to take the concentrations of the three main sex steroids into account.

We obtained a measure of state anxiety at every visit; however, because depression and catastrophising are considered trait measures these were only measured once. Given the strong influence of the hormonal milieu on the factors influencing the pain experience, it would have been useful to include other state measures of psychological or cognitive state to understand whether these have any mediating effect.

## Conclusions

We describe a state of “luteal analgesia,” during which the physiologically high levels of sex steroids seen after ovulation are associated with a specific reduction in the emotional component of pain and reduced brain activation in response to noxious stimuli. Given the availability of exogenous hormonal preparations, it may be possible to harness these benefits therapeutically. Similar studies in women with chronic pain and/or psychological distress are required to investigate the translational potential of these findings.

## Data availability

The datasets for this manuscript form part of a larger study for which analysis is still on-going and are thus not currently publicly available, however, they will be deposited in a publicly accessible repository once analysis is complete. Requests to access the datasets prior to deposition should be directed to Katy Vincent (katy.vincent@wrh.ox.ac.uk).

## Author contributions

KV, IT, JM, and SK designed the study. KV, CW, and CS performed the study and analyzed the data. KV drafted the manuscript. All authors contributed to the final version of the manuscript.

### Conflict of interest statement

The authors declare that the research was conducted in the absence of any commercial or financial relationships that could be construed as a potential conflict of interest.
